# Gene Specificity of Suppression of Transgene-Mediated Insertional Transcriptional Activation by the Chicken HS4 Insulator

**DOI:** 10.1371/journal.pone.0005956

**Published:** 2009-06-18

**Authors:** Romain Desprat, Eric E. Bouhassira

**Affiliations:** 1 Department of Medicine, Albert Einstein College of Medicine, Bronx, New York, United States of America; 2 Department of Cell Biology, Albert Einstein College of Medicine, Bronx, New York, United States of America; University of Florida, United States of America

## Abstract

Insertional mutagenesis has emerged as a major obstacle for gene therapy based on vectors that integrate randomly in the genome. Reducing the genotoxicity of genomic viral integration can, in first approximation, be equated with reducing the risk of oncogene activation, at least in the case of therapeutic payloads that have no known oncogenic potential, such as the globin genes. An attractive solution to the problem of oncogene activation is the inclusion of insulators/enhancer-blockers in the viral vectors. In this study we have used Recombinase-Mediated Cassette Exchange to characterize the effect of integration of globin therapeutic cassettes in the presence or absence of the chicken HS4 and three other putative insulators inserted near Stil, Tal1 and MAP17, three well-known cellular proto-oncogenes in the SCL/Tal1 locus. We show that insertion of a Locus Control Region-driven globin therapeutic globin transgene had a dramatic activating effect on Tal1 and Map17, the two closest genes, a minor effect on Stil, and no effect on Cyp4x1, a non-expressed gene. Of the four element tested, cHS4 was the only one that was able to suppress this transgene-mediated insertional transcriptional activation. cHS4 had a strong suppressive effect on the activation expression of Map17 but has little or no effect on expression of Tal1. The suppressive activity of cHS4 is therefore promoter specific. Importantly, the observed suppressive effect of cHS4 on Map17 activation did not depend on its intercalation between the LCR and the Map 17 promoter. Rather, presence of one or two copies of cHS4 anywhere within the transgene was sufficient to almost completely block the activation of Map17. Therefore, at this complex locus, suppression of transgene-mediated insertional transcriptional activation by cHS4 could not be adequately explained by models that predict that cHS4 can only suppress expression through an enhancer-blocking activity that requires intercalation between an enhancer and a promoter. This has important implications for our theoretical understanding of the possible effects of the insertion of cHS4 on gene therapy vectors. We also show that cHS4 decreased the level of expression of the globin transgene. Therefore, the benefits of partially preventing insertional gene activation are in part negated by the lower expression level of the transgene. A cost/benefit analysis of the utility of incorporation of insulators in gene therapy vectors will require further studies in which the effects of insulators on both the therapeutic gene and the flanking genes are determined at a large number of integration sites. Identification of insulators with minimal promoter specificity would also be of great value.

## Introduction

We and others have used mouse models to provide a proof of principle for gene therapy for the hemoglobinopathies [1]–[7]. Together, these studies have demonstrated that hematopoietic stem cells (HSCs) transduced by a lentiviral vector containing a globin gene, and transplanted to syngenic recipients can give rise to red blood cells expressing high levels of therapeutic globin chains. In the case of the sickle cell disease model, expression of the corrective globins in the transduced cells reached 52% of total hemoglobin production in 99% of the cells, a level that is sufficient to cure the disease.

These proofs of principle are a very significant advance in the field of gene therapy and were due to contributions by many investigators. Of particular importance was the development of vectors that can infect non-dividing mouse HSCs at high efficiency and of a better virus envelope that resists centrifugal forces, simplifying the production of concentrated viral stocks.

Since replication-defective gene therapy vectors integrate only once, it was generally believed that they would carry minimal risks of insertional mutagenesis in comparison with replication-competent viruses. Unfortunately, clinical trials for gene therapy of X-SCID in France and in England that successfully treated more than 80% of the patients have shown that the risk of mutagenesis is not insignificant [8], [9]. Insertional mutagenesis has therefore emerged as a major obstacle for gene therapy based on vectors that integrate randomly in the genome [10], [11].

### Mechanism of insertional mutagenesis

Studies in birds and mice have shown that two mechanisms account for the large majority of cases of oncogenesis mediated by retroviruses: the virus either encodes an oncogene or activates a proto-oncogene by insertional mutagenesis. The latter mechanism, almost always occurs by oncogene activation since very few tumor suppressors have been found near sites of viral integration in tumors induced by retrovirus (reviewed in [12]). Therefore, tumor suppressor inactivation is not a major insertional mutagenesis mechanism. As a result, reducing the risk of insertional mutagenesis for therapeutic globin genes, which have no known oncogenic potential, can, in first approximation, be equated with reducing the risk of oncogene activation.

### Mechanisms of gene activation by insertional mutagenesis

The mechanisms by which genes may be activated by insertional mutagenesis are multiple and include disruption of negative regulatory sequences, “hijacking” of a coding sequence by insertion of a viral promoter, and activation of a promoter by insertion of a viral enhancer. The latter mechanism is by far the most prevalent type of activation events found in animal tumors [12], probably because it requires the least precise insertion since enhancers can act at long distances.

Minimizing oncogene activation by enhancers carried by therapeutic vectors should therefore be an effective method of lowering the risks of insertional mutagenesis associated with gene therapy. Lessening the impact of each integration event involves understanding how highly expressed transgenes interact with their sites of integration, and then redesigning the vectors to decrease these interactions without drastically affecting transgene expression.

An attractive potential solution to the problem of oncogene activation is the inclusion of insulators in the viral vectors. Insulators are a complex class of cis-acting regulatory sequences with at least two separable activities. The first activity, often referred to as ‘enhancer-blocking activity’, prevents interaction between an enhancer and a promoter. The second activity, termed ‘barrier activity’, prevents the spread of condensed chromatin into a transcriptionally active region [13]. The best understood insulator in vertebrates is DNase I HS4 of the LCR of the chicken β-globin locus (cHS4) [14], [15]. cHS4 is located between the β-globin locus and an erythroid specific folate receptor gene [16]. Chung et al. have demonstrated that cHS4 has directional enhancer-blocking activity and barrier activity, since it protects from position effects in a colony assay, and prevents gradual silencing in cell culture [15], [17], [18].

Insulators have been shown to improve the levels of transgene expression, but the effects on the neighboring genes have not been adequately explored [19]–[25]. Theoretically, insertion of an insulator in the genome is expected to have complex effects since it should reduce the risk of insertional mutagenesis, but might also disrupt endogenous regulatory elements with unpredictable effects on gene expression. Understanding the mechanisms of action of insulators in greater details should help us predict their effects in a gene therapy context.

Recombinase Mediated Cassette Exchange (RMCE) is a powerful method to compare transgenes and gene therapy cassettes, because it can be used to site-specifically integrate transgenes in mammalian cells [26]. We previously created a number of RMCE integration sites termed RL1 to RL6 that can be used to study transgene expression in mouse erythroleukemia (MEL) cells. Integration of various cassettes at these sites of integration has yielded a wealth of information regarding the epigenetic mechanisms that control transgene expression, but we had not previously studied the effect of transgenes on neighboring genes. In collaboration with Walters et al., we reported almost 10 years ago, that as expected, the cHS4 insulator blocks enhancer-mediated suppression of silencing in K562 cells [27] but that the effects were complex and dependent on the site of integration. In the current study, we have evaluated the effects of cHS4 and of several other putative insulators on transgene expression and on the neighboring genes at one of these previously studied RMCE integration sites.

## Results

**Figure 1:**
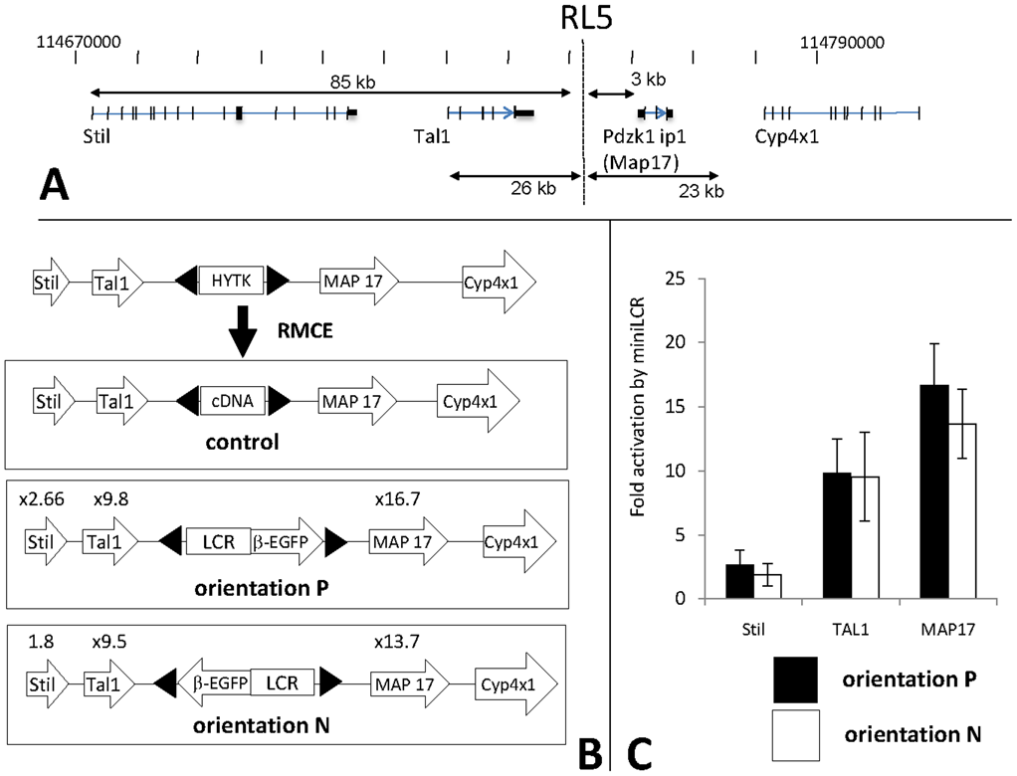
Insertion of cassette 234-β-EGFP activates genes near the RL5 integration site: A: Structure of the region around the RL5 integration site on chromosome 4. The integration site RL5 is located at Chr 4: position 114756771 (mouse build July 2007 (mm9) assembly). B: Schematic of the RMCE reactions. The numbers above the gene represent the average increase in levels of expression of the flanking genes after insertion of the 234-β-EGFP cassette at RL5. Both orientations are represented. The black triangle represents the two inverted Lox sites. Fold increases were calculated relative to the β-2-microglobulin gene and relative to the expression of the same gene when the control cDNA cassette was inserted at the same locus (see methods). C: Histogram summarizing the increase of the flanking genes (±standard deviation).

In order to evaluate the capacity of insulators to block insertional mutagenesis, we first inserted cassette 234-β-EGFP on chromosome 4 at site RL5 to determine if insertion of an expression cassette would activate the flanking genes. We chose site RL5 (Fig. 1a) for this analysis because it is located next to three important genes: RL5 is flanked on the left by two well-known oncogenes, Tal1, a transcription factor that regulates hematopoiesis [28], and Stil, the Scl/Tal1 interrupting locus gene which is involved in early embryonic development and in the control of cell proliferation. On the right, RL5 is flanked by Map17 (Pdzk1ip1), a widely expressed gene also known to be an oncogene [29], and by Cyp4X1, a gene of the cytochrome P450 family that is expressed at low levels in most tissues. The start sites of the Stil Tal1, Map17 and Cyp4X1 genes are respectively located 85, 24.6, 4.5 and 24 Kb from the RL5 site of integration.

We used cassette 234-β-EGFP as the reporter because we have studied it extensively in the past and because it is similar to cassettes that would be used for gene therapy. Cassette 234-β-EGFP contains the miniLCR driving the human β-globin promoter and the EGFP reporter. The miniLCR is a regulatory element that contains DnaseI Hypersensitive sites 2, 3 and 4 of the human β-globin Locus Control Region (LCR) a strong enhancer that is located 6 to 20 kb upstream of the ε-globin gene and that controls the expression of all β-like globin gen [30], [30]–[33,33]. We [34] and others [35], [36] have found that although the LCR is a powerful regulatory element, it is subject to silencing, since 150 kb YACs containing all the known regulatory sequences of the β-globin gene cluster are subject to both stable and variegating position effects in transgenic mice. The silencing is particularly acute with the truncated LCR derivatives that must be used in gene therapy vectors because of size constraints.

To determine the effect of cassette 234-β-EGFP on the flanking genes, we inserted it by RMCE at site RL5. The RMCE technique that we used is based on inverted identical Lox sites and leads to insertion of the cassettes in both possible orientations (termed non-permissive (N) and permissive (P) orientations). Since we have reported before that at some sites of integration, the orientation of the cassette plays a major role on transgene expression [37], we used PCR to characterize the orientation of the cassette in each clone and then selected clones with the cassette in each orientation for further analysis. To minimize clonal variations, at least three clones per orientation were studied.

After isolation of the targeted clones, we performed quantitative RT-PCR experiments and FACS analysis to assess expression of the transgene and of the four flanking genes. Five independent transfections performed on five different days were performed. As a control, we also inserted at RL5, a non-expressed cDNA transgene (Figure 1B). All quantitative RT-PCR results were normalized to expression of the β2-microglobulin gene. We then calculated for each flanking gene, the ratio of their expression in the presence of the 234-β-EGFP cassette and in the presence of the non expressed cDNA (see methods).

The results are summarized in Fig. 1c and showed that insertion of the 234-β-EGFP cassette in either orientation had a large enhancing effect on Map17 and Tal1, the two closest genes, and a smaller effect on Stil. Cyp4X1 is not expressed in MEL cells and was not reactivated by transgene insertion.

As expected, the EGFP reporter was expressed at easily detectable levels when cassette 234-β-EGFP was inserted at RL5.

These results suggested that RMCE at RL5 is a good experimental system to study insulator functions since insertion of a cassette containing regulatory elements that are necessary to express the β-globin genes in erythroid cells leads to activation of two of the four flanking genes.

**Figure 2:**
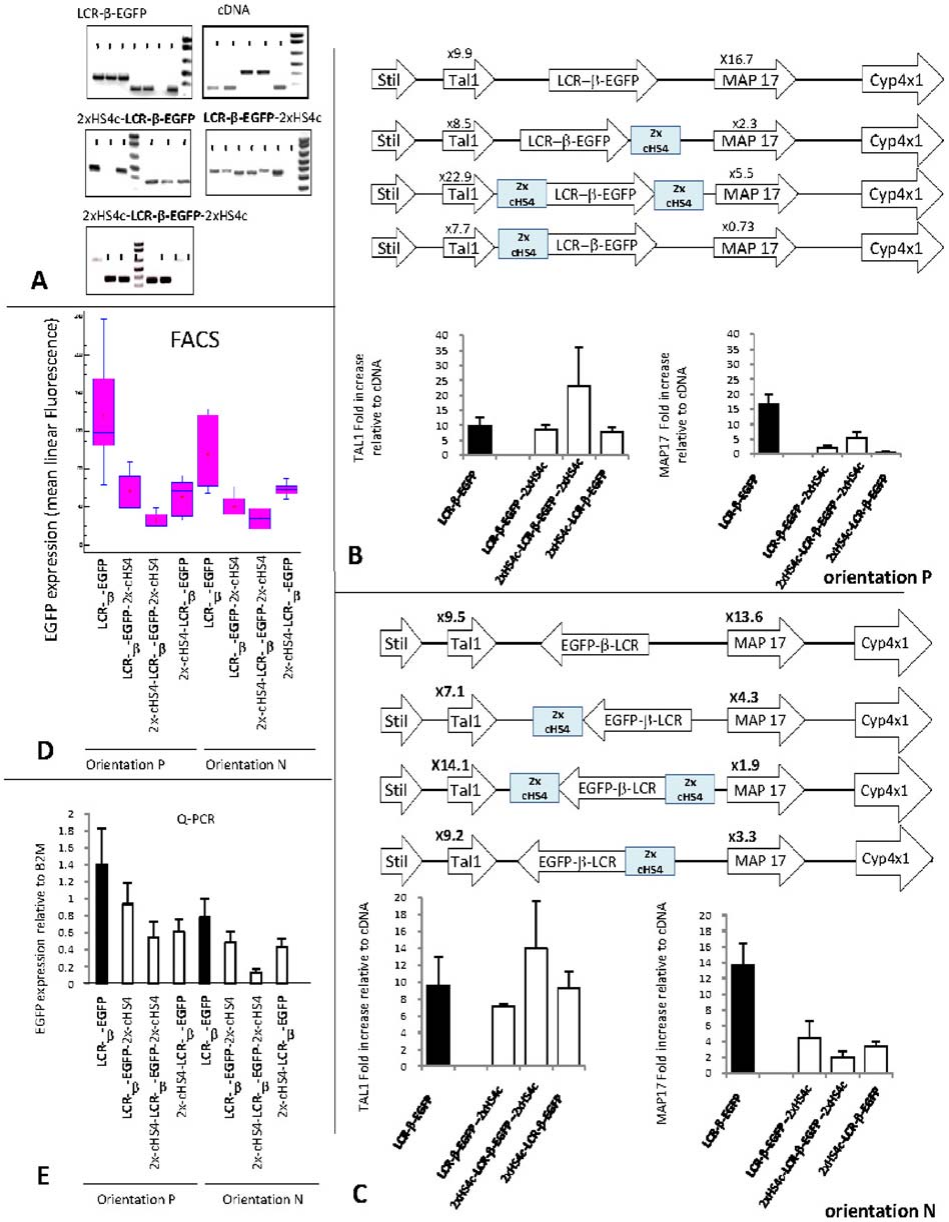
The cHS4 insulator block activation of Map17 but not Tal1. A: PCR analysis demonstrating insertion of 5 cassettes at RL5 in each orientation (see methods). At least 2 clones in each orientation are shown. B and C: Schematic of the structure of RL5 loci in the presence of the various tested cassettes in each orientation, and histograms illustrating the average activation (±standard deviation) of the flanking genes (relative to the control cDNA cassette). D: FACS Analysis: Whisker plots of the mean linear fluorescence of 5 to 15 clones containing cassette 234-β-EGFP flanked on either sides or on both sides by the 2.4 kb cHS4 insulator. Presence of one insulator decreases EGFP expression by more than 2-fold. Presence of 2 insulators has an even more pronounced effect. E: Histograms illustrating a Q-PCR analysis of EGFP expression of the clones analyzed by FACS in Figure 3A. The RT-PCR results are similar to the FACS results. EGFP expression was normalized to expression of the β-2-microglobulin gene. The effect of the insulator was independent of its location within the cassette and of the orientation of the cassette in the locus.

To test insulator function, we first focused on cHS4 since it is one of the better known insulators. We constructed three cassettes in which the 234-β-EGFP transcription unit is flanked by a 2.4 kb DNA segment containing two copies of the cHS4 insulator, either in 5′, in 3′ or on both sides. We chose to focus on this duplicated element because it has been shown to be more potent than smaller versions [25]. The insulated cassettes were then inserted at RL5 (Fig. 2a) and expression of the flanking genes was analyzed as above. Two to three independent transfections were performed for each cassette and at least three clones in each orientation were analyzed for each transfection.

The results are summarized in Fig. 2b and 2c and showed that presence of the insulator in all three cassettes had a dramatic effect on Map17 expression. In the presence of an insulator, levels of Map 17 expression were 5 to 10-folds lower that when cassette 234-β-EGFP without an insulator was inserted at RL5. Importantly and unexpectedly, the results were similar whether cHS4 was located on either side of the cassette or when it was present on both sides. Also unexpectedly, down regulation of Map17 occurred in all clones containing at least one cHS4, regardless of the orientation of the cassette in the RL5 locus. This suggests the cHS4 insulator can prevent activation of the Map17 promoter by the LCR but that this cis-acting negative regulatory activity does not require that the element be located between the enhancer and the promoter. Presence of two flanking copies of the insulator did not dramatically improve the “enhancer-blocking” effect.

Examination of expression of the Tal1 gene in the presence of the three insulator cassettes, revealed that the cHS4 had little or no detectable effect on Tal1 expression since that gene was over-expressed about 10-fold when compared to the cDNA control cassettes whether or not an insulator was present. This lack of effect was observed with all three insulated cassettes tested, suggesting that suppression of the transgene-mediated insertional activation enhancer activity of cHS4 is promoter specific, because it can block activation of Map17 but cannot block activation of Tal1.

Examination of EGFP expression by flow cytometry showed that presence of an insulator decreased the expression of the transgene 2 to 3-folds when the insulator was present on one side, and 3 to 4-folds when the insulator was present on both sides (Fig. 2d and 2f). Quantitative RT-PCR confirmed and extended these results by showing that, as expected, the down regulation of the miniLCR-β-EGFP cassette occurs at the transcriptional level.

To determine if other insulators would be more efficient than cHS4 in this assay, we then tested three other elements: human HS5 (hHS5), human HS4 (hHS4) and human gamma satellite repeats. Human HS5 is the ortholog of cHS4 and has been shown to have developmental stage specific, CTCF-mediated insulator activity in cell culture and in transgenic mice [38], [39]. Human HS4 is not an insulator and has enhancer activity. It was included in this study because we have found that it could slow down gene silencing at some chromosomal location (data not shown). Finally, we tested a 1.8 kb γ-satellite repeat that has recently been found to have strong insulator activity by the Larionov lab (personal communication, [40]). Gamma-satellite DNA has been identified in the pericentromeric regions of human chromosomes 8, X, and Y [41], [42], [43] and is composed of tandem arrays of 220 bp GC-rich repeating units, usually forming 10–200 kb clusters flanked by α-satellite DNA. In this study we have used a 1.8 kb DNA fragment, termed Gamma8 (G8) that is composed of eight 220 bp diverged monomers and that was cloned from the centromere of chromosome 8 and that contains multiple CTCF and Ikaros binding sites.

**Figure 3:**
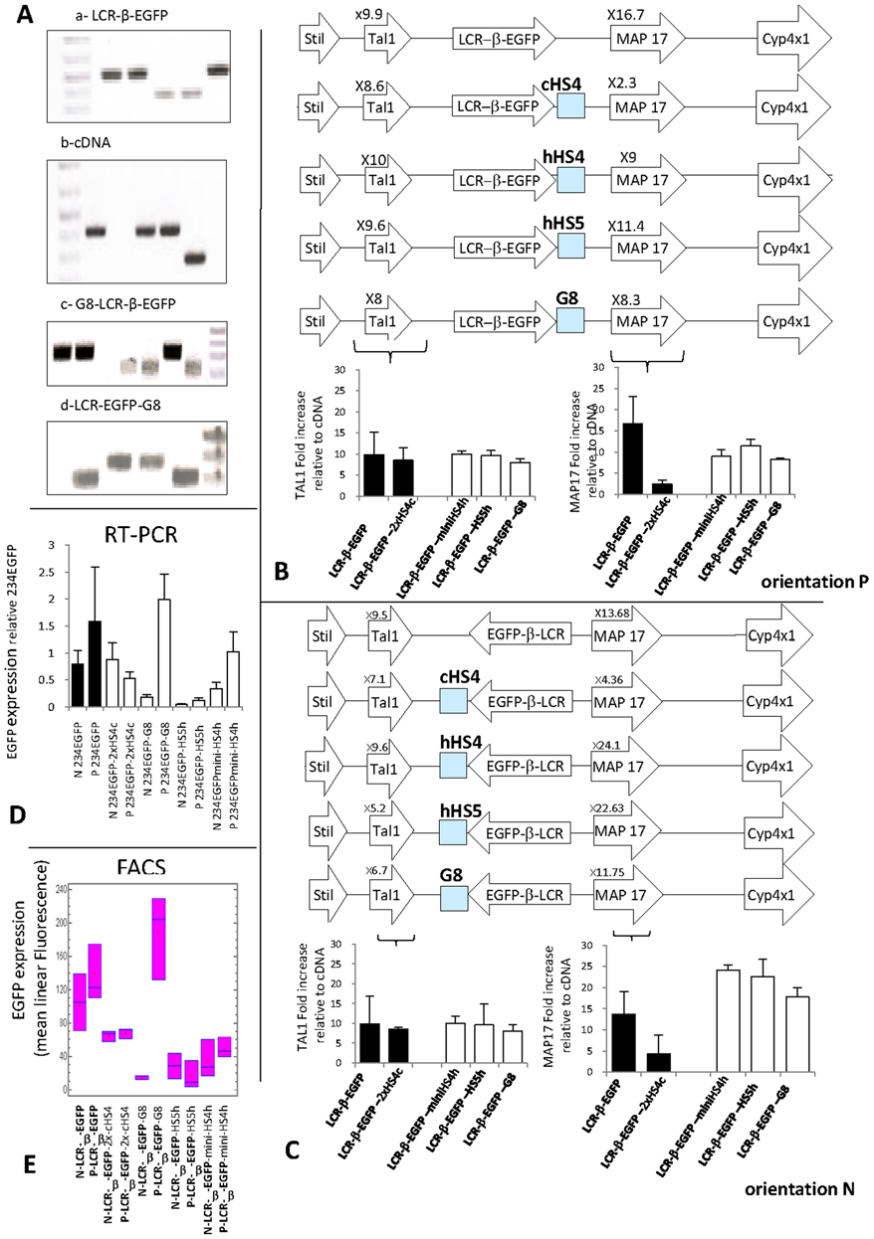
Insertion of hHS4, hHS5 and hG8 repeat do not block activation of Tal1 and Map17. A: PCR analysis demonstrating insertion of the various cassettes. B and C: Diagram illustrating the structure of the RL5 region after insertion of the various cassettes and histograms summarizing Q-RT-PCR determinations of the average fold increases (±standard deviation) of the flanking genes relative to the cDNA control cassette. The three cassettes tested had minimal effects on expression of Tal1 and Map17. The black bars represent the fold increase of the 234-β-EGFP cassette with and without cHS4 which was used as a control in this experiment. D and E: FACS and Q-RT-PCR analyses of EGFP expression (see Figure 3D and 3E) when cassettes 234-β-EGFP plus hHS4, hHS5 or G8 were inserted at RL5. Levels of expression in the presence of HS5, HS4 and G8 are respectively lower or higher than the controls both at the protein and mRNA levels.

Cassettes containing hHS5, hHS4 and G8 sequence cloned 3′ of our 234-β-EGFP, as well as controls including the uninsulated 234-β-EGFP cassette, the cDNA reference cassette and the 234-β-EGFP-cHS4 cassettes were inserted at RL5 (Fig. 3a) and expression of the flanking genes was assessed as described above.

Fig. 3B and 3C illustrate the results which showed that none of these regulatory elements had any consistent cis-acting negative regulatory or enhancer-blocking effect on activation of the Map17 or the Tal1 gene by the miniLCR. On the contrary, all three elements seemed to have a small enhancing effect on Map17 when they were inserted in the N orientation. Analysis of EGFP expression by FACS (Fig. 3D and 3E) revealed that expression of EGFP was down regulated by presence of hHS5 and hHS4. The effects of presence of the G8 repeats were more complex (see below).

To further examine the effect of G8 on expression of the flanking genes, we then inserted at RL5, two cassettes in which the G8 putative insulator was cloned on either sides and on both sides of the 234-β-EGFP cassette. Again, G8 had no effect on expression of the flanking genes (data not shown). We concluded from these experiments that although the G8 mini-satellites are rich in CTCF binding sites it has no enhancer-blocking activity.

**Figure 4:**
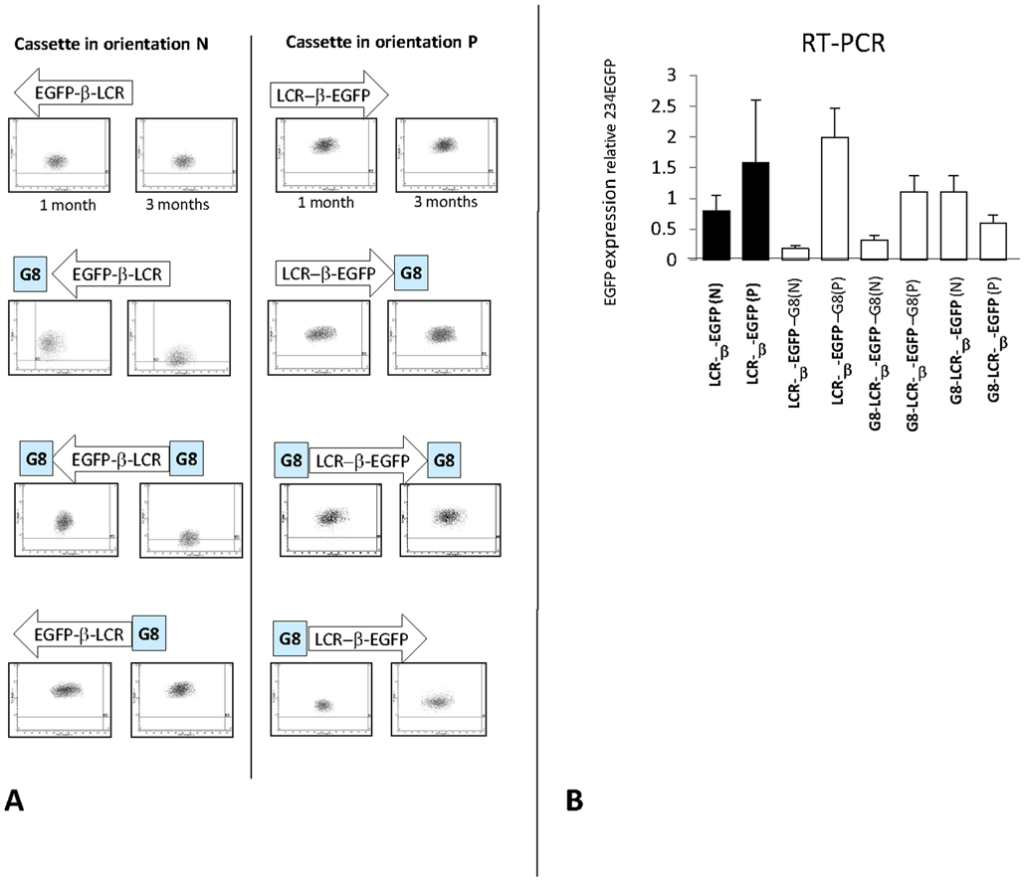
Orientation-dependent silencing of β-EGFP expression in the presence of the G8 repeats. A: Dot-plots illustrating EGFP expression of representative clones one or three months after RMCE. X-axis: forward-scatter; y-axis: FL-1 (EGFP) fluorescence. The horizontal line represents the level of auto-fluorescence of non-tranfected control MEL cells. Presence of one copy of G8 3′ of EGFP, or of two flanking copies of G8 caused silencing of the transgene in the N but not in the P orientation. B: Q-RT-PCR analysis of EGFP expression demonstrating that the silencing induced by the G8 repeats occurs at the mRNA level.

We previously reported that expression is extremely stable at RL5 since transgenes inserted at this site are not silenced even after continuous culture for more than 40 passages [37]. Clones with all of the cassettes tested in this report were passaged for at least three months. As expected, no silencing was observed in either orientation when the miniLCR-β-EGFP cassette alone or in the presence of cHS4, hHS4 and hHS5 was inserted at RL5 (data not shown). However, presence of the G8 satellites repeats led to complete silencing after less than three months of culture when the G8 repeats was between the Tal1 and the EGFP gene, but not when it was between the EGFP and Map17 gene (Fig. 4A). Quantitative RT-PCR analysis demonstrated that the silencing occurred at the mRNA level (Fig. 4B). This demonstrates that while the G8 repeat does not have any detectable enhancer blocking in our assay, it does have a strong epigenetic effect on expression.

## Discussion

Insertion of transgenes at random sites of integration for gene therapy or to create transgenic animals has long been complicated by position effects which results either in silencing of the transgene over time or to varying levels of gene expression. Position effects are likely caused by the random juxtaposition of regulatory elements present in the transgene and in the flanking sequences. We and others have previously studied in great details expression of transgenes in the presence of various regulatory elements using RMCE and a variety of others methods but relatively few studies have focused on the effects of transgene insertion and expression of the flanking genes. This question has come to the forefront in the gene therapy field because of several reports of insertional mutagenesis.

From a theoretical point of view, insertion of an insulator or an enhancer blocker at a random site in the genome is a double edged sword since on one hand it should minimize activation of flanking genes by enhancers present in the transgene, but on the other hand, it might perturb expression of the flanking genes by interrupting communication between endogenous regulatory sequences. RMCE, which allows highly efficient integration of transgenes at predefined sites of integration is particularly well suited to address these questions.

In this study, we have tested the effect of insertion of eight different cassettes on expression of the four genes nearest to the RL5 site of integration. The results clearly showed that integration of the miniLCR-β-EGFP had a dramatic activating effect on Tal1 and Map17, the two closest genes, a minor effect on Stil, and no effect on Cyp4x1, a non-expressed gene. The effects of transgene insertion on expression of the neighboring genes are expected to be complex but it is likely that activation of the Tal1 and Map17 genes is largely caused by the miniLCR. This suggests that the miniLCR can activate at least three promoters (Tal1, Map17 and β-globin) at the same time.

The results with cHS4 are striking. This regulatory element has a strong suppressive effect on the expression of Map17 but has little or no effect on expression of Tal1.

Importantly, the suppression of Map17 activation did not depend on the intercalation of cHS4 between the LCR and the Map 17 promoter. Rather, presence of one or two copies of cHS4 anywhere within the transgene was sufficient to almost completely eliminate the transgene-mediated insertional activation of Map17.

This result differs from previous reports [14] in which the enhancer-blocking activity of cHS4 was defined as the “suppression of enhancer action by an element positioned between and enhancer and a promoter”. Formally, the suppressive activity of cHS4 that we have detected can therefore not be called an enhancer-blocking activity. In any case, our results clearly show that cHS4 affects gene expression in a way that is more complex than previously believed since the suppression of transgene-mediated insertional activation of this element is promoter specific and is not well described by simple directional models of enhancer-blocking activity.

The differences between our results and previous studies are likely due to the fact that the previous studies were based on simple systems in which one enhancer and one promoter were tested, while in this report multiple promoters and enhancers were located in close proximity (see below).

Regulation of the Tal1 locus has been studied in details by a number of labs. The Tal1 gene can be expressed from three different promoters and is regulated by at least eight regulatory regions which are used differentially in different tissues [44]. It has also been suggested that the Tal1 and Map17 genes are co-regulated [44].

Interestingly, interaction between the LCR, the insulator and these regulatory regions leads to relatively constant outcomes regardless of the orientation of the cassette in the locus and of the linear arrangement of the regulatory elements within the transgene: insertion of the miniLCR-β-EGFP cassette leads to activation of Map17 and Tal1, and insertion of cHS4 blocks activation of MAP17 but not of Tal1. We propose that at RL5, the transgenic and the endogenous regulatory elements might interact in three dimension and form similar structures regardless of their linear arrangements. This hypothesis is compatible with models that suggest that transcription occurs in factories [45] and with the models that suggest that the LCR can form chromatin hubs [46], [47]. The mechanism of hub or loop formation has been studied in some details fo the Drosophila gypsy insulator and at several loci mammalian loci including the β-globin and IgF2/H19 region.

In Drosophila, loop formation is believed to involve the nuclear lamina and the Su(Hw) complex [48], [49]. In mammals, CTCF has been shown to mediate the formation of a loop involving the imprinting control region (ICR) and DMR1 regions that would restrict access of the Igf2 to its enhancer [50], [51]. CTCF has recently been shown to nullify the activity of the LCR on the β-globin gene by mediated loop formation between the LCR and ectopically placed HS5 that was intercalated between the LCR and the β-globin gene in transgenic mice [52]. The mechanism by which CTCF might cause loop formation has recently been clarified by studies that showed that CTCF often co-localize with cohesin, that CTCF help position cohesion on DNA and that cohesion is required for some of the known transcriptional effects of CTCF [53]. Recently, Wallace and Felsenfeld proposed that the enhancer-blocking activity of cHS4 might be a corollary of a wider role of this class of elements in organizing large scale structures within the nucleus [14]. Our observations support this hypothesis.

However, the linear arrangement of cis-acting regulatory elements is in some context a critical determinant of gene expression. For instance, we previously reported that transgenes exchanged at RL4 and RL6, two sites of integration that were generated at the same time as the RL5 site [37], were silenced over a 6 to 12 weeks period in an orientation-specific manner. Similarly, we have found in the current study that presence of the G8 repeats induces silencing at RL5 in an orientation-dependent manner. We therefore propose that specific linear arrangements of regulatory elements are incompatible with long term replication of the epigenetic information necessary to keep the chromatin in an open conformation. Stochastic progressive silencing may be due to the formation, with a relatively high probability, of suppressive three-dimensional structures (hubs) which induce the formation of a permanently closed heterochromatin. The mechanisms that destabilize the stable replication of the epigenetic information and lead to permanent silencing are not completely understood but may involve transcriptional interference and change in the timing of DNA replication as we previously reported at other sites [54], [55], [56].

From a gene therapy point of view, our results suggest that simple models of insulator and enhancer blocking function cannot completely predict the effect of an insulated vector on the genes near the site of integration. We demonstrated that cHS4 could be used as an enhancer-blocker to prevent activation of Map 17, but not of Tal1. We also show that cHS4 decreased the level of expression of our transgene. Therefore, the potential benefit of preventing activation of Map17 is partly negated by the lower expression level of the transgenes. This down-regulation of the transgene might be an idiosyncrasy of the RL5 locus since several studies have shown that on average presence of an insulator increases expression levels. The finding that human HS4 and HS5 and the G8 repeats can also decrease expression of the 234-β-EGFP transgene suggests that down-regulation of EGFP when cHS4 is 3′ of the cassette is not specific to that element. A recent study using RMCE to insert transgenes inside an intron of the Lmo2 locus suggests that insulators decrease insertional activation of this gene and would therefore be beneficial [57].

Nevertheless, our study suggests that insertion of an insulator in gene therapy vectors might be associated with a cost in terms of level of transgene expression and might not block activation of all genes. A similar conclusion was reached by a previous study of the same insulator in a different cell line [58]. A complete cost/benefit analysis of the utility of incorporation of insulators in gene therapy vectors will require further studies in which the effects of insulators on both the therapeutic gene and the flanking genes are determined at a large number of loci. Identification of insulators with minimal promoter specificity would also be of great value.

## Methods

### RMCE

RMCE reactions were performed as previously described [37]. Briefly, RL5 cells grown in DMEM containing 10% fetal bovine serum and 5% CO_2_ were selected for resistance to Hygromycin for at least 1 week, and used for transfection while in exponential growth phase. For each electroporation, 2×10^6^ cells were spun down and resuspended in 400 µl total volume of cell culture medium containing 25 µg of Cre expressing plasmid (SSR) and 100 µg of shuttle (targeting) plasmid (1∶4). The cells were then electroporated at 230 Volts and a capacitance of 1180 uf (Gene Pulser Biorad) and transferred very slowly to 6-well plates containing 10 ml of pre-warmed media. 48 h after the electroporation cells were plated into 96-wells plates with Gancyclovir at 1.1 mg/ml at a concentration of 1000 to 10,000 per well. Clones were isolated 10 days later and expanded for further analysis.

The orientation of the cassettes in each clone was determined by PCR using the primers described in Table 1. For each cassette studied, two primer pairs (one for each orientation) composed of one primer annealing to the cassette and the other to the flanking DNA at RL5 were designed so that a product would only be created by the PCR if a site specific insertion had occurred.

**Table 1:** Primers to determine orientation.

plasmid	PCR Primer	Description	NP	Orientation
p232	848–425	EGFP234	P	Tal1-<--EGFP-betaP-234-MM17
p232	848–437	234EGFP	N	Tal1-432-betaP-EGFP-->-MM17
p272	848–437	noP-EGFP	N	Tal1-EGFP-->-MM17
p272	848–937	noP-EGFP	P	Tal1-<--EGFP-MM17
p313	848–1044	234EGFPmini-HS4h	N	Tal1-432-betaP-EGFP-->-HS4 Mini h-MM17
p313	848–425	234EGFPmini-HS4h	P	Tal1-MiniHS4h-<--EGFP-beta promoter234-MM17
p346	848–938	LCR	P	Tal1-HS4-3-2-MM17
p415	1038–848	234EGFP-HS4c	N	Tal1-432-betaP-EGFP-->-HS4chicken-MM17
p415	848–1038	234EGFP-HS4c	N	Tal1-432-betaP-EGFP-->-HS4chicken-MM17
p415	848–425	HS4-EGFP234	P	Tal1-<--HS4chicken-EGFP-betaP-234-MM17
p415	850–425	234EGFP-HS4c	N	Tal1-432-betaP-EGFP-->-HS4chicken-MM17
p417	848–1038	234EGFP-HS5h	N	Tal1-432-beta promoter-EGFP-->-HS5 human-MM17
p417	848–425	234EGFP-HS5h	P	Tal1-HS5 human-<--EGFP-beta promoter234-MM17
p423	848–1038	2xHS4c-EGFP-234	N	Tal1-432-betaP-EGFP-->-2xHS4chicken-MM17
p423	848–425	234EGFP-2xHS4c	P	Tal1-<-2xHS4chicken-EGFP-betaP-234-MM17
p423	848–437	234EGFP-HS4c	N	Tal1-432-betaP-EGFP-->-2xHS4chicken-MM17
p423	850–425	234EGFP-2xHS4c	N	Tal1-432-betaP-EGFP-->-2xHS4chicken-MM17
p430	848–1042	2xHS4c-234EGFP-2xHS4c	N	Tal1-2xHS4chicken-432-betaP-EGFP-->-2xHS4chicken-MM17
p430	848–1070	2xHS4c-234EGFP-2xHS4c	N	Tal1-2xHS4chicken-432-betaP-EGFP-->-2xHS4chicken-MM17
p450	848–1021	cDNA	N	Tal1-cDNA-MM1
p450	848–1025	cDNA	P	Tal1-ANDc-MM17
p461	848–1038	EGFP-234-2xHS4c	P	Tal1-<--EGFP-betaP-432-2xHS4c-MM17
p461	848–1070	2xHS4c-234-EGFP	P	Tal1-<--EGFP-betaP-432-2xHS4c-MM17
p461	848–437	2xHS4c-234-EGFP	N	Tal1-2xHS4c-432-betaP-EGFP-->-MM17
p465	1038–1072	HS4 chicken	P	Tal1-5′HS4c3′-MM17
p465	848–1075	HS4 chicken	N	Tal1-3′HS4c5′-MM17
p473	848–1140	234EGFP-G8	N	Tal1-G8-EGFP-betaP-234-MM17
p473	848–425	234EGFP-G8	P	Tal1-432-betaP-EGFP-G8-MM17
p475	848–1138	G8-234EGFP-G8	N	Tal1-G8-EGFP-betaP-234-G8-MM17
p475	8481140	G8-234EGFP-G8	P	Tal1-G8-234-betaP-EGFP-G8-MM17
p475	848–1140	G8-234EGFP-G8	P	Tal1-G8-234-betaP-EGFP-G8-MM17
p476	848–1138	G8-234EGFP	P	Tal1-EGFP-betaP-234-G8-MM17
p476	848–437	G8-234EGFP	N	Tal1-G8-432-betaP-EGFP-MM17

Each RMCE transfection included the test cassettes plus the cDNA cassette and the 234-β-EGFP cassette. The latter two cassettes were used as normalization controls for each experiments.

### Plasmids

The components of the plasmids used in this study are described in Table 2. The sequences of all plasmids are available on request. The cre expression plasmid and the 234-β-EGFP cassettes have been previously described in [37]. The 2.4 kb cHS4 double insulator fragment was a generous gift from Dr Felsenfeld (NIH). The 1.8 kb fragment containing the Gamma8 repeats was a generous gift of Dr. Larionov (NIH, NCI). A portion of the cDNA of the human ADAMTS13 gene was used as an “inert” control cassette. We used a mammalian cDNA rather than a prokaryotic, non-mammalian, or artificial DNA segment for this control because insertion of DNA fragment with abnormal GC content had a fairly large effect on expression at the RL5 locus (data not shown).

**Table 2:** DNA segment inserted in the various cassettes used in this study.

Name	Size (bp)	Accession Number	Location	Freeze genomic
Human HS4	1247		Chr 11:5265030-5266276	Human May 2006
Human HS5	2652	L22754	chr:11:5267271-5269928	Human May 2006
2× Chicken HS4	1211	U78775	chr1:199422885-199424103	Chicken May 2006
Gamma 8	1932		8q11.1	Human

The cHS4 insulator used in this study contained 2 tandem duplicated copies of U78775.

RT PCR: RT-PCR reactions were done on a light-Cycler (Roche) using 75 ng of RNA per primer pairs and using the QuantiTect SYBR Green RT-PCR Kit. (Qiagen, Cat Number: 204243). RT-PCR conditions for all primers were: 50°C for 1200 sec (RT reaction) (slope 20°C/sec), 95°C for 900 sec (polymerase activation) (slope 20°C/sec), followed by 36 cycles of 94°C for 15 sec (slope 20°C/sec), 58°C for 30 sec,72°C for 30 sec (slope 2°C/sec)}. The Tm of the product was then evaluated as follows: 95°C for 10 sec (slope 20°C/sec), 65°C for 10 sec (slope 20°C/sec),95°C for 0 sec (slope 0.1°C/sec). RT-PCR primers are described in Table 3.

**Table 3:** primer used for RT-PCR.

Name	gene	Sequence primer 1	Sequence primer 1
EB 732/733	Beta2M	CTACTCGGCGCTTCAG	TGTTCGGCTTCCCATT
EB740/741	EGFP	AACTACAAGACCCGCG	CGGCCATGATATAGACGT
EB881/EB 882	MM17	CTGGGAGCACAGTGATGATCATTGGAAA	GTGTGCTGCGGACCCTG
EB 869/870	TAL	CAACAACAACCGGGTGAAGAG	ACTTCATGGCAAGGCGGA
EB871/872	SIL	CCGTGAATGCGCCAAGACC	ACACTGGCATGATCCACTTTCTG
EB 877/878	CYT P450	GGGAGAAGATGTGGACCACTCAT	ATATGATTTCACCAAGTTCAAATGTTGAA

Expression of EGFP, Tal1, Map17, Stil1 and Cyp4x1 was estimated:

1. By calculating the Delta Ct(gene of interest) (for instance Ct(Tal1)-Ct(B2M)) for clones containing the cassette of interest (for instance 234-b-EGFP).
2. By calculating the DeltaCt(control cassette) (for instance Ct(tal1-Ct(B2M)) for clones containing the cDNA control cassettes.
3. And finally by calculating the fold increase: 2exp^(-Delta Ct(gene of interest)-DeltaCt(control cassette))^.

All PCR were performed in triplicate on at least two independent series of three clones.

### FACS analysis

Flow cytometry analysis was performed 1, 2 and 3 months after electroporation on a BD FacsCalibur (Detector,Voltage,AmpGain,Mode:FSC,E1,6.31,Lin;SSC,379,1.65,Lin;FL1, 400,1, log;FL2,444,1,log.Compensation: FL1-0.5% FL2, FL2-38.6% FL1). 50,000 cells were washed once in PBS with 1% FBS and resuspended in PBS containing 1 µg/ml of Propidium Iodide. EGFP mean linear fluorescence was normalized to the florescence of the 234-β-EGFP cassette that had been used as a control in the transfection and that had been cultured in parallel with the tested cassette. Inclusion of this control was necessary to minimize variation of the FACS measurements since no external calibration was used for the FacsCalibur.
